# Prevalence of Hypertension and Associated Factors in an Indigenous Community of Central Brazil: A Population-Based Study

**DOI:** 10.1371/journal.pone.0086278

**Published:** 2014-01-28

**Authors:** Geraldo F. Oliveira, Teresinha R. R. Oliveira, Adauto T. Ikejiri, Mariela P. Andraus, Tais F. Galvao, Marcus T. Silva, Maurício G. Pereira

**Affiliations:** 1 Clinical Medicine Department, Federal University of Grande Dourados, Dourados, Brazil; 2 Faculty of Health Sciences, Federal University of Grande Dourados, Dourados, Brazil; 3 Faculty of Medicine, University of Brasilia, Brasília, Federal District, Brazil; 4 Getulio Vargas University Hospital, Federal University of Amazonas, Manaus, Amazonas, Brazil; 5 Faculty of Medicine, Federal University of Amazonas, Manaus, Amazonas, Brazil; University of Sao Paulo Medical School, Brazil

## Abstract

**Objective:**

The aim of the present study was to assess the prevalence of hypertension and cardiovascular risk factors among the native indigenous of Jaguapiru village in Dourados, Mato Grosso do Sul, Brazil.

**Method:**

A cross-sectional, population-based study was conducted with adult indigenous aged 18 years or more. The subjects' blood pressure was measured twice, and the mean of the two measurements was calculated. Body weight, height, capillary blood glucose and waist circumference were measured. Pregnant women, individuals using glucocorticoids, and non-indigenous villagers and their offspring were excluded. Multivariate regression analyses were conducted on the socio-demographic and clinical independent variables. Interactions between independent variables were also tested.

**Results:**

We included 1,608 native indigenous eligible to the research. The prevalence of hypertension was 29.5% (95% CI: 27–31.5), with no significant difference between the genders. For both men and women, diastolic hypertension was more common than systolic hypertension. The prevalence of hypertension was higher among obese, diabetic, and older participants, as well as those who consumed alcohol, had a lower educational level, or had a family history of hypertension. There was no association between hypertension and tobacco smoking or family income.

**Conclusion:**

Hypertension among the indigenous from Jaguapiru village was similar to the prevalence in the Brazilians, but may have a more negative effect in such disadvantaged population. The associated factors we found can help drawing prevention policies.

## Introduction

Indigenous populations worldwide exhibit a lower life expectancy and poorer health status compared with non-indigenous populations [1]. There is an important segment of indigenous population in Brazil. The Brazilian indigenous population currently comprises 817,000 individuals distributed across 688 communities [2]. These individuals represent approximately 0.4% of the Brazilian population and occupy almost 12% of the national territory [2]. According to estimates, 36,2% of them live outside indigenous villages [2].

Changes in habits, the disintegration of the socio-cultural environment, the severance of links to the land, and crises of identity contribute to the increase in the prevalence of chronic diseases among the Brazilian indigenous population [3]. In addition, high susceptibility to diseases, poor living conditions, and restricted access to healthy food among indigenous peoples, combined with inadequate and inefficient healthcare services, make the situation even more dramatic [3]. Although there is vast diversity among indigenous peoples, there are many similarities in their health status, diseases, and the determinants of their health.

Chronic non-infectious diseases are the most common cause of death worldwide. Cardiovascular disorders are responsible for 48% of these deaths, and the hypertension is involved in the origin, progression, and occurrence of future cardiovascular disease [4], [5]. For instance, the prevalence of hypertension is high among indigenous peoples in North America and Australia [6]–[8]. Previous studies investigated the prevalence of diabetes mellitus, impaired glucose tolerance, and metabolic syndrome in the indigenous of Jaguapiru village, in central Brazil [9], [10]. Hypertension, however, was not deeply investigated in this population.

The aim of the present study was to assess the prevalence of hypertension among adult indigenous from the village of Jaguapiru and the relationship between the prevalence of hypertension and socioeconomic factors and known cardio-metabolic risk factors. This study in particular might add additional information to the overall prevalence of hypertension which is essential to assess the burden of this disease in Brazil.

## Methods

### Study design and context

The present study was a cross-sectional, population-based study of the prevalence of hypertension and associated factors in Jaguapiru indigenous village in the municipality of Dourados, Mato Grosso do Sul, in the central region of Brazil. The study was conducted from January 2009 to July 2011. The Dourados indigenous reservation is located close to an urban area and is home to the *Guarani*, *Kaiowá*, and *Terena* ethnicities [11]. The population density of this area is high, and the socioeconomic conditions in the communities deteriorated along with the local ecosystem: the native forest and the wild fauna have fully disappeared, affecting the indigenous' means of subsistence [11].

The study was approved by the Research Ethics Committee of the University Center of Grande Dourados (UNIGRAN; no. 197/07) and the National Commission of Research Ethics (CONEP; no. 14453). All participants signed an informed consent form.

### Participants

At the onset of the study, the total population of Jaguapiru village consisted of 5,727 indigenous who resided in 1,255 dwellings. We considered eligible native indigenous (not multiracial) of both genders aged 18 years or more. As pregnancy may influence the blood pressure, pregnant women were not eligible. We aimed to include all eligible subjects (universe), thus a sample size calculation was not necessary.

### Data sources

On the first visit to the participants' homes, they were interviewed about the socioeconomic status, dietary habits, physical activity, self-reported use of alcohol and tobacco, age, gender, occupation, personal and family history of diabetes mellitus and hypertension, educational level, and health conditions.

The indigenous' ages were recorded using their *Fundação Nacional do Índio*, (FUNAI, the Brazilian public organ for indigenous affairs) identity card. All socioeconomic variables, alcohol and tobacco consumption were self-reported. No scales or objective measurements were employed.

Undergraduate medical students at the Federal University of Grande Dourados who were enrolled in the Diabetes League completed the data forms. All researchers received special training to ensure uniformity in the data collection process.

### Blood pressure measurement

Arterial pressure was measured by two trained researchers (ATI, TRRO) using an aneroid sphygmomanometer (BD®) for adults, which was calibrated every 3 months. The sphygmomanometer cuff was placed on the right arm of the participants while they were in a seated position with their legs uncrossed and their feet on the ground. Blood pressure was measured after a resting period of 10 minutes, with a 5 minute interval between them. The final arterial pressure value recorded was the arithmetic mean of 2 consecutive measurements. When the systolic or diastolic pressures exhibited a difference greater than 4 mmHg, a third measurement was performed, and the most extreme measurement was disregarded. The participants were considered to have hypertension if they had a systolic pressure ≥140 mmHg and/or a diastolic pressure ≥90 mmHg on examination, if they had receive a previous diagnosis of hypertension, or if they used anti-hypertensive drugs on a regular basis [12].

### Blood glucose assessment

On the second visit, one researcher (GFO) measured the capillary blood glucose after a 12-hour fasting period using a glucometer (Accu-Chek®) that was calibrated on a weekly basis and fast-reading reagent strips (glucose-oxidase). Blood glucose levels from 70 to 99 mg/dL were considered normal, and values from 100 to 125 mg/dL were defined as prediabetes. The participants in the latter category were subjected to an oral glucose tolerance test, performed by one researcher (GFO).

The individuals with blood glucose levels between 126 and 199 mg/dL had their capillary blood glucose measured again at a later date to confirm the diagnosis of diabetes mellitus. The diagnoses of diabetes mellitus and prediabetes were based on the criteria established by the American Diabetes Association [13].

### Anthropometric measurements

Body weight (in kilograms) was measured while the participants wore their usual clothes and no shoes, using a standardized digital weighing scale. The scale was calibrated on a monthly basis and was capable of measuring up to 180 Kg.

Height (in centimeters) was measured while the participants stood barefoot using a portable aluminum stadiometer capable of measuring heights ranging from 80 to 220 cm. Body mass index (BMI) was calculated as weight in kilograms (kg) divided by height in meters squared (m^2^). Obesity was considered as BMI ≥30 kg/ m^2^.

Waist circumference (in centimeters) was measured using an inelastic tape measure placed on the midpoint between the lower margin of the last rib and the upper part of the iliac crest while the participants were standing erect. The normal upper limit of waist circumference was established as 90 cm for men and 80 cm for women; such cut off points were based in the International Diabetes Federation, since specific scales to our study population is not available [14].

Anthropometric measurements were collected by two trained professionals who worked on this study (ATI, TRRO).

### Statistical analysis

The qualitative variables are presented as absolute (n) and relative (%) frequencies and the quantitative variables are presented as means and standard deviations. Hypertension was the dependent variable.

Univariate Poisson regression analysis with robust variance was applied to the sociodemographic and clinical independent variables. The variables with p-values <0.25 were selected for inclusion as co-variables in the multiple regression analysis [15]. The modified version of Akaike's information criterion (QIC_u_) was used to select multivariate models, and the model with the lowest QIC_u_ value was chosen [16]. Multicollinearity among the independent variables was assessed using the variance inflation factor, and in addition, the significance of the interactions among the independent variables was tested. Point and interval prevalence ratios were calculated relative to the selected model. With regard to the associations among the variables, the level of significance was established at 5% (p-value <0.05).

The following interactions were tested: obesity and alcohol consumption, alcoholism and family history of hypertension, and diabetes and obesity.

## Results

**Figure 1:**
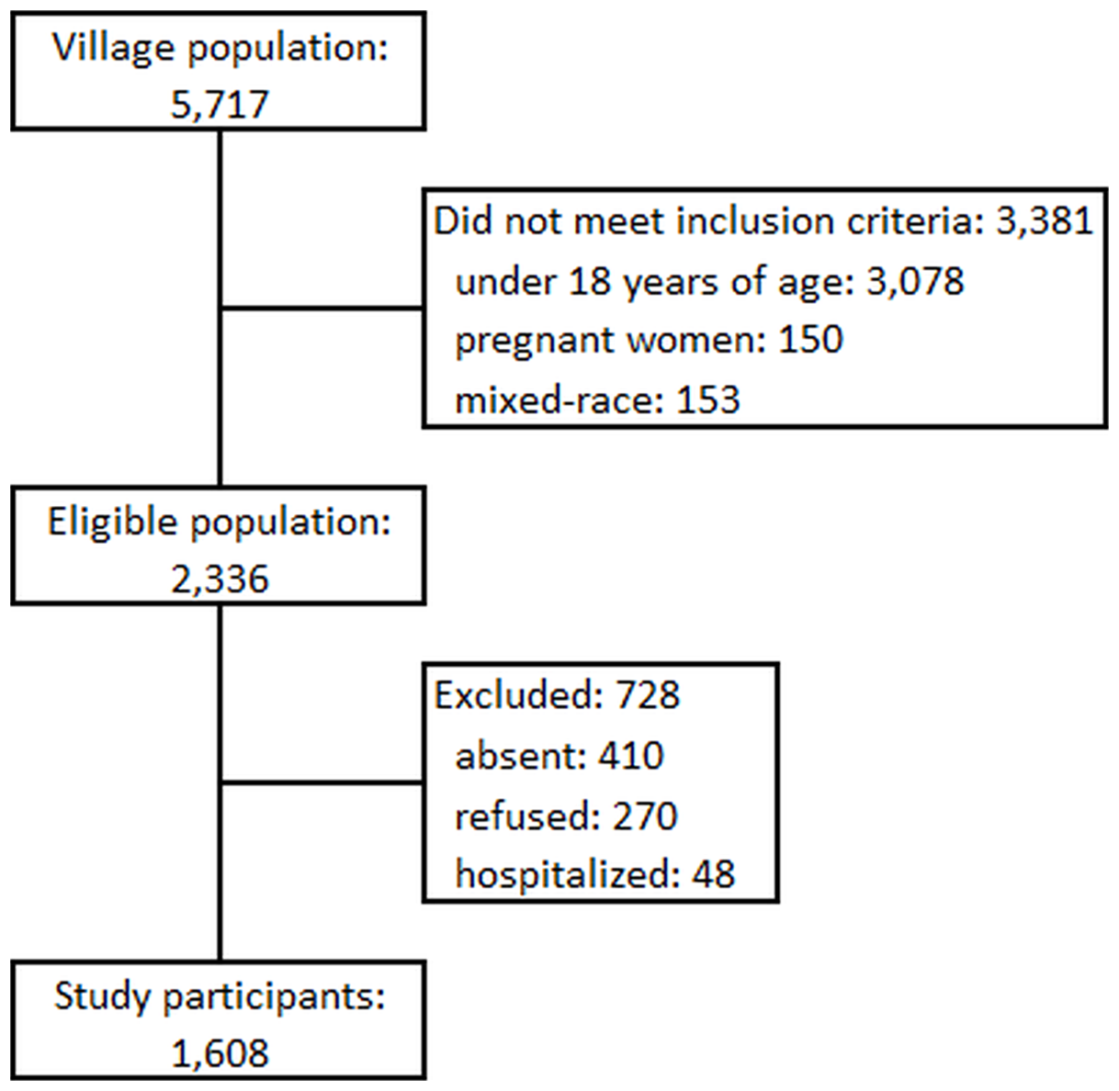
The process of inclusion of participants in the study.

A total of 1,608 Brazilian native indigenous were included in the present study, what accounted for 69% of the whole eligible population from the village (Figure 1). Most of the participants were young: 38.5% of the participants had less than 30 years old, and only 21.7% were older than 49 years of age. The participants' educational level was low, and only 2.1% had higher education (Table 1). The monthly family income was less than US$ 315 for 41.1% of the families. Sugarcane cultivation was the main economic activity for the male indigenous (35.3%). Tilling their land was a secondary form of economic activity (17.2%). Household chores were the main activity performed by the women, followed by jobs at public schools and healthcare centers on the Indigenous Reservation (data not shown).

**Table 1:** Socioeconomic, demographic, and clinical characteristics of the study participants.

Characteristic	Male (n = 729)	Female (n = 879)	Total (n = 1,608)
Age, years, mean (SD)	37.4 (14.7)	38.0 (15.5)	37.7 (15.1)
Schooling, %			
No elementary education	73.8	71.8	72.7
Elementary education	16.9	16.3	16.5
Intermediate education	7.0	10.0	8.6
Higher education	2.3	1.9	2.1
Family income, minimum wage, % ^a^			
<1	NA	NA	41.1
1 to 2	NA	NA	33.7
2 to 3	NA	NA	18.1
≥ 3	NA	NA	7.0
Smoker, %	26,2	13,1	19,0
Consumption of alcohol, %	31,1	8,9	19,0
Participation in physical activity, %	49.4	24,0	35.5
Height, cm, mean (SD)	165.7 (6.8)	153.6 (5.8)	159.1 (8.7)
BMI in kg/m^2^, mean (SD)	26.2 (4.1)	27.6 (5.0)	27.0 (4.7)
Family history of hypertension, %	41.6	52.6	47.8
Blood pressure in mmHg, mean (SD)			
systolic	126.2 (19.2)	125.0 (21.1)	125.5 (20.3)
diastolic	81.6 (12.6)	80.3 (14.3)	80.7 (13.6)
Diabetes, %	2.9	7.8	5.8
Hypertension, % ^b^	28.5	30.3	29.5
Hypertension for age in years, %			
18–29	13.4	8.3	10.6
30–39	24.4	25.7	25.1
40–49	37.3	43.3	41.1
50–59	54.1	72.6	63.3
≥ 60	54.3	54.9	54.6

Notes:

SD, standard deviation;

NA, not available.

a, Minimum wage at time of study: 315 USD. Not included in the male/female columns because it reflects the whole family income.

b, Blood pressure systolic ≥140 and/or diastolic ≥90 mmHg.

Among the known risk factors for cardiovascular disease, old age, diabetes and alcohol consumption were the most prevalent (Table 2). Among the obese individuals, 44.8% had hypertension versus 19.6% of the individuals with normal weight (Table 2).

**Table 2:** Associations between demographic, socioeconomic and clinical variables and the prevalence of arterial hypertension, based on Poisson multiple regression analysis (n = 1.608).

Variable	% of Hypertension	Prevalence ratio	95% CI	p-value
Age, years				
18 to 29	10.63	1.00	-	-
30 to 39	25.06	2.03	1.53–2.70	<0.01
40 to 49	41.07	3.21	2.42–4.25	<0.01
50 to 59	63.31	4.59	3.50–6.01	<0.01
≥60	54.64	4.24	3.22–5.58	<0.01
Education				
No elementary education	33.96	2.53	1.08–5.91	0.03
Elementary education	15.79	1.66	0.69–4,00	0.26
Secondary education	22.30	2.20	0.90–5.36	0.08
Higher education	11.76	1.00	-	-
Family history of hypertension				
No	27.05	1.00	-	-
Yes	32.16	1.23	1.08–1.41	<0.01
Weight				
Normal	19.61	1.00	-	-
Overweight	30.18	1.07	0.85–1.34	0.57
Obesity	44.77	1.43	1.13–1.82	<0.01
Diabetes				
No	27.01	1.00	-	-
Yes	69.15	1.39	1.16–1.65	<0.01
Alcohol consumption				
No	28.70	1.00	-	-
Yes	32.79	1.38	1.16–1.64	<0.01
Abdominal waist circumference				
Below the limit	18.28	1.00	-	-
Above the limit	36.88	1.25	0.99–1.57	0.06

The prevalence of hypertension was 29.5% (95% CI: 27%–31.5%), and there was no statistically significant difference between men and women. For both men and women, diastolic hypertension (25.3%) was more common than systolic hypertension (19.9%). Among the individuals previously diagnosed with hypertension, only 19% had normal blood pressure values (data not shown). The prevalence ratio (PR) of hypertension was 4.2 (95% CI: 3.2–5.6) for those between 60 and 69 years of age (Table 2).

Table 2 displays the variables that remained significant after adjustment. There was a statistically significant positive association between the prevalence of hypertension and age (p<0.01), a family history of hypertension (p<0.01), obesity (p<0.01), diabetes (p<0.01) and alcoholism (p<0.01). There was a statistically significant negative association between the prevalence of hypertension and higher education (p = 0.03). Smoking and family income did not exhibit any association with the prevalence of hypertension.

The analyses of the interactions between the cardio-metabolic risk factors and the prevalence of hypertension showed positive and significant associations between alcohol consumption and obesity (PR  = 1.93; 95% CI: 3.80–6.68), a family history of hypertension and alcohol consumption (PR  = 1.71; 95% CI: 1.36–2.14), and obesity and diabetes mellitus (PR  = 1.93; 95% CI: 1.47–2.53) (data not shown).

## Discussion

Almost a third of the indigenous population had high blood pressure. The prevalence of hypertension was higher among obese, diabetic, people that had a family history of hypertension and older participants. Most of the present study's results align with several epidemiological studies that have revealed an association between blood pressure levels and clinical, demographic, socioeconomic, and lifestyle factors [17]–[23]. However smoking and family income did not exhibit an association with the prevalence of arterial hypertension. The family income range of indigenous communities is not similar to the range that modern societies exhibit. A positive association between obesity and hypertension, which has been shown in non-indigenous populations [24], [25] and in Mexican indigenous [26], was confirmed in the present study: 44.8% of the participants with hypertension were obese versus 19,6% of the participants with normal blood pressure. Because BMI was the parameter used to diagnose obesity, the results should be assessed with caution because most of the men are manual workers who might exhibit well-developed muscle mass, which falsely influence BMI. In other words, high BMI values might reflect a large amount of muscle mass rather than a large amount of fat [27].

With regard to smokers, it is possible that the lack of an association might reflect changes in habits induced by a previous diagnosis of hypertension. Smokers may have a mild reduction in blood pressure, mainly related to decreased BMI and to the vasodilator effect of cotinine, the main metabolite of nicotine [28].

Due to the diversity of the Brazilian indigenous communities, the reported prevalence of hypertension among these peoples has varied widely (from 4.8 to 64%) in past studies [29]–[32]. The hypertension rates found in Australian indigenous communities (from 24.9 to 51.7%) are higher than the rate recorded in the present study [7]. In addition, in a cohort of individuals older than 45 from 13 indigenous communities in the Midwestern US who were followed for 10 years, the prevalence of hypertension varied widely (from 24.9 to 44.9%), increased with age, and was similar to the prevalence in the overall US population [33]. These variations in the prevalence of hypertension in the various populations might be attributable to variations in weight, sedentary lifestyles, blood glucose disorders, dietary habits, and genetic factors in each community. Furthermore, the procedure for diagnosing blood pressure has a key role, along with the sampling design in each study [34].

The prevalence of arterial hypertension found in the present study is similar to the prevalence rate of 22.7% that was identified in a recent survey conducted in all of the Brazilian state capitals with individuals in the same age range [35]. The prevalence of arterial hypertension is also similar to the 20% reported in a systematic review on the prevalence of arterial hypertension in Brazil that was conducted during the late 1990s [36]. The rate reported in the former study must be analyzed cautiously because the data were collected over the telephone exclusively from individuals formally diagnosed with arterial hypertension [35]. This prevalence is similar to the 30% prevalence estimated in a recent study of the Brazilian population older than 30 years old [37].

The present study has some limitations. The study included subjects from only three indigenous peoples (*Guarani*, *Kaiowá*, and *Terena*) who were in different stages of assimilation into Western culture and exhibited varying epidemiological profiles. This population may not be representative of all the Brazilian natives. It has been widely reported in the literature that the risk factors for arterial hypertension vary as a function of place and ethnicity [33]. Second, there were a greater number of female participants in the study, possibly because it was easier to recruit and follow-up women, as the men were frequently unavailable due to their temporary work schedule and stay at a nearby village. This may not be significant as there were no gender differences observed in the prevalence of hypertension. Third, the cross-sectional nature of the study provides weak evidence of causation. Nevertheless, the present study might prompt future longitudinal studies on the effects of changes in lifestyle on the health of the local population.

Some measurements of present study's variables may have not been optimal: Reagent strips were used for measuring blood glucose, which is a less precise method than serum sample measurements [38]; The blood pressure result was derived from arithmetic mean of two consecutive measurements; which was held in the own house of the subject. These procedures were taken to reduce bias and avoid “white coat hypertension”, however, the increase in blood pressure due to being observed may be included in this outcome [39]. To set normality for waist circumference, overweight, and hypertension, the cutoffs points used derived from other populations, since limits defined within the scrutinized population were not available.

As per our knowledge, the present study is the first population-based study to assess the prevalence of arterial hypertension in indigenous communities of the Brazil Central. Previous studies focused in effects of nutritional transition in this population [9], [10].

In conclusion, the present study found that the prevalence of hypertension among the adults indigenous of Jaguapiru village was similar to the prevalence among the Brazilian population. However, the burden of hypertension in the indigenous population, with multiple shortages of resources and healthcare accessibility, is probably higher than in the general population and may help explaining lower life expectancy in this ethnicity. Our data also urges for early detection and appropriate management of hypertension among the indigenous population of Brazil.
